# Breast Cancer Knowledge Among Amish and Mennonite Women

**DOI:** 10.1007/s13187-024-02452-7

**Published:** 2024-05-14

**Authors:** Melissa K. Thomas, Ashleigh Gilligan, Jenna Lawson, Ted Lau

**Affiliations:** 1 Department of Primary Care, Ohio University Heritage College of Osteopathic Medicine, Athens, OH USA; 2 Athens, OH USA; 3 Ohio University Heritage College of Osteopathic Medicine, Dublin, OH USA

**Keywords:** Breast Cancer Knowledge, Amish and Mennonite women, Rural health beliefs

## Abstract

Breast cancer is the most common cancer diagnosis for women in the USA and ranks second in cancer-related deaths. Disproportionately higher breast cancer rates can be found in rural and Appalachian regions due to several social drivers of health, including poverty, access to healthcare, and lack of culturally sensitive health education. Amish and Mennonite communities, religious groups with distinct cultural practices and beliefs, experience lower mammography screening and higher breast cancer mortality rates (among Amish women). This study focuses on knowledge about breast cancer and causes of cancer among Amish and Mennonite women. A total of 473 women participated in the study at 26 separate women’s health clinics throughout Ohio, consisting of 348 Amish and 121 Mennonite women, the largest study conducted on breast cancer knowledge spanning dozens of communities. Statistically significant differences were found in total knowledge scores between Amish and Mennonite women (*r*_pb_ = .178, *n* = 466, *p* = .007), with Amish women having lower scores and stronger beliefs in myths associated with breast cancer cause and symptoms (*χ*(1) = 7.558, *p* = .006). Both groups often provided scientifically accurate descriptions of cancer etiology. The majority of participants underestimated breast cancer risk, highlighting the need for culturally appropriate health education programs that consider numeracy and health literacy. By implementing targeted interventions and fostering partnerships with community stakeholders using a multifaceted approach that incorporates cultural sensitivity, community engagement, and collaboration, significant progress can be made towards reducing breast cancer disparities and improving health outcomes.

## Introduction

Breast cancer ranks the most commonly diagnosed cancer among women in the USA (with the exception of skin cancers) and also ranks second in cancer-related deaths among women [1]. Breast cancer mortality rates tend to be even higher among women from racial/ethnic minority groups and those residing in economically disadvantaged areas. Several barriers to care contribute breast cancer disparities, including inadequate medical communication with the community, difficulty in accessing healthcare services, racial disparities, and lower rates of health insurance coverage. Additionally, limited access to breast cancer screenings due to financial constraints can result in later-stage diagnoses, especially among uninsured and under-insured individuals [2].

Rural regions like the Appalachian areas of Pennsylvania, Kentucky, and Ohio face unique challenges in breast cancer screening and diagnosis. These regions exhibit lower participation rates in breast cancer screening programs and a higher incidence of late-stage breast cancer diagnoses [3]. A broader review of breast cancer in rural areas has identified elevated breast cancer rates compared to urban areas. One specific population within these rural settings, the Amish community, also experiences distinctive healthcare barriers. The Amish are a religious group with roots in the Anabaptist movement and sixteenth-century Protestant Reformation interpretations from Switzerland who migrated to the USA in pursuit of religious freedom during the eighteenth and nineteenth centuries [4]. Each Amish community maintains a distinct identity with a common core set of beliefs but varying regulations, practices, and interpretations. The Amish population has been steadily growing, doubling approximately every 22 years and is now spread across 568 geographical locations in North America, represented in 32 of the 50 states [5]. As of 2021, Ohio is home to the second-largest Amish settlement, located in the Holmes County Area, estimated to comprise over 36,000 people.

The Amish face healthcare barriers stemming from both their rural locales and their cultural practices. Language barriers exist, as many Amish primarily communicate in a dialect called Pennsylvania Dutch and German within their communities, using English only for interactions with non-Amish individuals [6]. Limited formal education, typically ending after eight grade, has contributed to lower literacy levels compared to their non-Amish counterparts in the same regions. [7] The selective acceptance of some technologies and the rejection of many others that both physically and symbolically represent too much connection to the outside world may lead to an even greater lack of cancer information than that which already exists for most other rural populations in underserved regions [4].

Previous research has shown lower mammography rates among Amish women compared to non-Amish women [8]. While the Amish community does not exhibit higher breast cancer incidence rates, it does face significantly higher mortality rates among those diagnosed with breast cancer [9]. Though research on Amish women remains limited, breast cancer was the leading cause of death in Amish women under the age of 60 living in two of the world’s largest Amish settlements in Ohio. Lower screening rates contribute to later-stage diagnoses and higher mortality rates, possibly compounded by a lack of breast cancer education and the prevalence of cancer-related myths within the community [2].

The purpose of this study was to assess knowledge about breast cancer and what causes cancer among Amish and Mennonite women throughout Ohio. Each community is united by core values but have different practices and specific beliefs, making the large data set of different Amish Communities in Ohio a closer representation of the Amish as a whole rather than a specific community’s beliefs.

## Materials and Methods

Our cross-sectional mixed-method study was a sequential explanatory design [10] that consisted of a self-administered questionnaire followed by a one-on-one interviewer-administered session of open-ended questions related to knowledge about breast cancer. Data was collected at women’s health clinics throughout all of Ohio between October 2017 and March 2019 for Amish and Mennonite women living mostly in Ohio but included residents of neighboring states in Indiana, Kentucky, and Pennsylvania. The clinics were organized by the community-led program called Project Hoffnung (German for Hope): The Amish and Mennonite Breast Health Project [11]. A total of 26 women’s health clinics were scheduled in some of the most rural parts of the state serving Amish and Mennonite communities. Clinics included a mobile mammography unit that provided reduced-cost or no-cost mammograms. All clinics were held at local community churches with close proximity to Amish and Mennonite communities so that participants could travel by horse and buggy, walk, or ride their bicycles if so desired. The churches provided space with electricity for registration, education, and a mobile office for a nurse practitioner to provide clinical breast exams, Pap tests, pelvic exams, and one-on-one patient consultation. A portable exam table and medical supplies were transported to each clinic site. When possible, local health care organizations (e.g., hospitals, health departments) attended specific clinics to offer additional health care services and introductions to nursing staff and resources. In addition to a contracted nurse practitioner, team members included a designated Amish Community Health Worker, a breast educator, the Program Coordinator (a certified Community Health Worker), the Founding Director (also a Certified Community Health Worker), and local community volunteers.

The survey consisted of five sections: demographic information and past clinic participation history, mammography screening history, health history, and seven knowledge questions adapted from Champion’s Health Belief Model survey [12]. The knowledge questions can be found in Table 1. Past clinic and screening history included questions about family history of breast cancer, survivor status, services received (such as education, mammograms, clinical breast exams), insurance coverage, health insurance status, anticipated services for the following year, and mammogram history (whether they have had one, frequency, and timing of their last mammogram). The survey assessed participants’ perceived health status and existing health problems, including conditions like high blood pressure, diabetes, heart disease, and cancer. We also asked if the participant experienced any symptoms suggestive of breast cancer and actions taken in response to those symptoms.

**Table 1:** Characteristics of survey participants by religion

Demographics	Amish (*n* = 348) *n* (percent)^ϯ^	Mennonite (*n* = 121) *n* (percent)^ϯ^	p*
Age category (in years)			.664**
18–29	8 (2.3)	0 (0.0)	
30–39	7 (2.0)	2 (1.6)	
40–49	90 (26.2)	32 (26.0)	
50–64	175 (51.0)	72 (58.5	
65 +	63 (18.4)	17 (13.8)	
Marital status			.647
Married	183 (89.3)	92 (87.6)	
Single	15 (7.3)	6 (6.1)	
Widowed	7 (3.4)	6 (5.7)	
Other	0 (0.0)	1 (1.0)	
Number of years in the program			.340**
1–5	229 (66.8)	88 (71.5)	
6–10	83 (24.2)	29 (23.6)	
11–15	27 (7.9)	6 (4.9)	
16 +	4 (1.2)	0 (0.0)	
History of cancer			
No	212 (62.4)	77 (63.1)	
Yes	128 (37.6)	45 (36.9)	
Breast cancer survivor			.568
No	331 (99.1)	122 (100.0)	
Yes	3 (0.9)	0 (0.0)	
Insurance			
No	208 (97.2)	98 (93.3)	
Yes	6 (2.8)	7 (6.7)	
Level of education			< .001
8th grade	194 (91.5)	70 (70.7)	
8th grade + GED up to HS	18 (8.5)	29 (29.3)	

^ϯ^Percentages are based on nonmissing values

^**^Significance values listed for correlation statistics on continuous variables (age and number of years in program)

*p<.05

After completion of the survey, participants met with an interviewer in a private room to answer six open-ended questions related to knowledge, attitudes, and beliefs surrounding breast cancer. The questions included the following: What is your main reason for being here today?, Do you know anybody who does not want to get a mammogram, If yes, why not?, What do you say to someone who does not want to get a mammogram?, What is cancer?, and What do you think of when you hear the word cancer? A recorder was present for each session who wrote down all responses on paper. Participants also received a tailored education intervention before receiving screening and medical care at the clinic. A member of the Amish or Mennonite community was present at each clinic to address any cultural/linguistic support, but all participants were fluent in English as a second language. Results pertaining to attitudes, beliefs, and the education intervention will be reported in subsequent publications. The study was approved by the Institutional Review Board at Ohio University.

### Statistical Analysis

Data was collected on paper for both self- and interviewer-administered sessions. All data was entered into an Excel document and imported into a statistical software program (IBM SPSS version 25 or higher) [13]. The open-ended responses were transcribed from written notes and verified by both the main interviewer and recorder for accuracy. Since the data was collected over a 1-year period, it was possible for the same woman to participate more than one time for annual screenings; therefore, we deleted any duplicate surveys for the same person and only included the first survey in the dataset. We used an inductive coding approach by consensus coding that included three separate coders (MT, AG, TL) who met weekly to compare results, discuss themes, and finalize the codebook. Frequency data on each qualitative code was calculated for each survey participant in order to compare the responses of Amish and Mennonite communities. A total knowledge score was calculated by assigning one point for each correct response to the seven-item knowledge questionnaire.

Descriptive statistics were calculated for each variable. Chi-square test and Fisher’s exact test were used to measure associations between categorical variables, and odds ratios with confidence intervals were calculated for binary outcomes. Correlation statistics were calculated for continuous variables including total knowledge score, age, and number of years participating in the screening program. The statistical analyses were performed with a *P* value less than or equal to 0.05 using two-tailed tests.

## Results

A total of 473 women participated in the survey, consisting of 348 Amish women and 121 Mennonite women, with a mean age of 55 years (Table 1). Out of the total participants, 278 (89.2%) reported being married, 21 (6.6%) were single, and 13 (4.1%) were widowed. A total of 173 (37.7%) women reported having a family history of breast cancer, with 44 (36.7%) identifying as Mennonite and 129 (37.4%) as Amish (Table 1). Among all participants, 334 (72.4%) were returning participants (and within 2 years of previous visit), while 131 (27.6%) were new to the program or had not participated in over 2 years. There were no statistically significant differences between the Amish and Mennonite groups with respect to key demographic variables.

The mean total knowledge score was found to be 5.03 (out of a possible total score of 7), with Amish women having a mean score of 4.91 and Mennonite women having a mean score of 5.36. There was a statistically significant correlation between participation level and the total knowledge score, *r*_pb_(38) = 0.358, *p* = 0.023, with returning participants having higher total knowledge scores than new participants (5.46 ± 1.23 versus 3.96 ± 1.30). Level of participation (new vs returning) accounted for 22.1% of the variability in total knowledge scores.

In response to the question about whether there is pain associated with the early stages of breast cancer, 436 (92.8%) women answered correctly (no pain). Specifically, 317 (92.3%) Amish and 119 (99.2%) Mennonite women answered correctly (*P* = 0.006) (Table 2). When asked if bumping or bruising breasts caused breast cancer, 381 (82.0%) women answered correctly (False). Amish women were more likely to incorrectly believe in the relationship between bumping or bruising and breast cancer with 272 (79.8%) answering correctly versus 106 (87.6%) of Mennonite women (*P* = 0.055).

**Table 2:** Comparison of breast cancer knowledge of the Amish and Mennonite study groups

Variable	OR	CI	Amish (*n* = 348) *n* (percent)	Mennonite (*n* = 121) *n* (percent)	p*
On the average, how many women will get breast cancer sometime during their lives?	1.396	.908, 2.145			.128
Correct			199 (58.0)	81 (65.9)	
Incorrect			144 (42.0)	42 (34.1)	
Who do you think is more likely to get breast cancer?	1.009	.665, 1.533			.965
Correct			200 (58.3)	72 (58.5)	
Incorrect			143 (41.7)	51 (41.5)	
Can bumping or bruising the breasts lead to breast cancer?	1.793	.982, 3.272			.055
Correct			272 (79.8)	106 (87.6)	
Incorrect			69 (20.2)	15 (12.4)	
Is pain always associated with the early stages of breast cancer?	9.968	1.338, 74.272			.006*
Correct			313 (92.3)	120 (99.2)	
Incorrect			26 (7.7)	1 (.8)	
How often should a women over 40 have a mammogram?	1.136	.654, 1.974			.650
Correct			281 (81.9)	103 (83.7)	
Incorrect			62 (18.1)	20 (16.3)	
The amount of radiation used in a mammogram is about the same as you would get living in your home for a year. This amount is considered	1.306	.647, 2.639			.456
Correct			304 (88.6)	112 (91.1)	
Incorrect			39 (11.4)	11 (8.9)	
On the average, the risk of a women getting breast cancer sometime during her life is	1.647	1.084, 2.503			.019*
Correct			118 (34.4)	57 (46.3)	
Incorrect			225 (65.6)	66 (53.7)	

*p<.05

Two questions referred to the risks of developing breast cancer. Question 1 used a ratio format to ask lifetime risk (1 in 8, 1 in 24, or 1 in 100), with 284 (60.0%) women answering correctly (1 in 8). Question 7 used low, medium, or high responses to estimate risk, with only 178 (37.6%) women answering correctly (high). Amish women were more likely to answer breast cancer risk as low when compared to Mennonite women (14.3% versus 8.1%, respectively) (*P* = 0.034). Again, past participation had a significant impact on estimating breast cancer risk, with 165 (48.4%) of past participants stating high versus only 13 (9.9%) of new participants (*P* < 0.001).

A key finding of the study highlights the importance of addressing the perception of breast cancer risk. When asked about the risk of breast cancer over a woman’s lifetime in terms of ratio responses (Q1), 60% of respondents correctly answered 1 in 8. However, when asking the same question in terms of degree of risk (low, medium, high) (Q7), only 38% correctly answered high. There was a significant positive association between the number of years participating in the program (i.e., repeated education intervention sessions) and both risk questions (Q1, *P* < 0.001; Q2, *P* = 0.017). The more years a woman participated in the clinic, the more likely she was to correctly answer questions related to breast cancer risk.

Qualitative responses were analyzed to identify common themes regarding the perceived causes of breast cancer. The most frequent response, given by 345 (73.1%) participants, was “don’t know,” with Mennonite participants less likely to respond with the statement (OR = 0.395, 95% CI = 0.253, 0.615, *P* < 0.001) (Table 3). Mennonite women were more likely to attribute the cause of breast cancer to cells (37.4% versus 17.2%; *P* < 0.001) and genetics (27.6% versus 17.5%; *P* = 0.016). From a more physical and emotional standpoint, Mennonite women were also more likely to mention treatment when describing cancer (27.6% versus 17.8%; *P* = 0.020) and pain and suffering (11.4% versus 3.5%, *P* = 0.001).

**Table 3:** Qualitative themes associated with cancer and breast cancer etiology among Amish and Mennonite study groups

Variable	OR	CI	Amish (*n* = 346) *n* (Percent)	Mennonite (*n* = 120) *n* (Percent)	p*
Cells	3.343	2.090, 5.347			< .001*
Yes			52 (15.2)	46 (37.4)	
No			291 (84.8)	77 (62.6)	
Something that eats	2.246	.959, 5.264			.057
Yes			13 (3.8)	10 (8.1)	
No			330 (96.2)	113 (91.9)	
Disease	1.557	.849, 2.857			.150
Yes			282 (82.2)	108 (87.8)	
No			61 (17.8)	15 (12.2)	
Growth/Mass	1.428	.841, 2.423			.186
Yes			52 (15.2)	25 (20.3)	
No			291 (84.8)	98 (79.7)	
Lumps	.927	.330, 2.605			.885
Yes			15 (4.4)	5 (4.1)	
No			328 (95.6)	118 (95.9)	
Genetics	1.802	1.111, 2.922			.016*
Yes			60 (17.5)	34 (27.6)	
No			283 (82.5)	89 (72.4)	
Hormones					.099**
Yes			10 (2.9)	8 (6.5)	
No			333 (97.1)	115 (93.5)	
Treatment	1.766	1.090, 2.861			.020*
Yes			61 (17.8)	34 (27.6)	
No			282 (82.2)	89 (72.4)	
Poor Health	1.123	.482, 2.621			.788
Yes			20 (5.8)	8 (6.5)	
No			323 (94.2)	115 (93.5)	
Stress					.456**
Yes			8 (2.3)	1 (0.8)	
No			335 (97.7)	122 (99.2)	
Environment					.214**
Yes			8 (2.3)	6 (4.9)	
No			335 (97.7)	117 (95.1)	
Lifestyle	1.330	.665, 2.658			.419
Yes			28 (8.2)	13 (10.6)	
No			315 (91.8)	110 (89.4)	
Just happens	1.532	.597, 3.933			.372
Yes			13 (3.8)	7 (5.7)	
No			330 (96.2)	116 (94.3)	
Pain/suffering	3.543	1.591, 7.891			.001*
Yes			12 (3.5)	14 (11.4)	
No			331 (96.5)	109 (88.6)	
Bump/bruise					.284**
Yes			16 (4.7)	3 (2.4)	
No			327 (95.3)	120 (97.6)	
Don’t know	.395	.253, .615			< .001*
Yes			270 (78.7)	73 (59.3)	
No			73 (21.3)	50 (40.7)	
Other					.735**
Yes			9 (2.6)	2 (1.6)	
No			334 (97.4)	121 (98.4)	

^**^Fisher’s exact test performed when expected cell count < 0

*p<.05

While not statistically significant, Amish women did mention bumping or bruising more often than Mennonite women when describing causes of breast cancer (4.7% versus 2.4%, respectively). Interestingly, Amish women would often mention something not true such as a myth and then follow-up with a statement that they did not believe it. For example, one Amish woman stated, “Some people think if you bump your breast, but I don’t think that.” Another Amish woman noted, “I used to think a bump, but I bumped mine and nothing came of it, so can’t be that.” Amish women also had a tendency to mention others’ points of view about causes of breast cancer, with one woman responding “I’m not sure they know it but some people say if you bump and bruise it, it will lead to breast cancer”.

## Discussion

The purpose of this study was to examine knowledge about breast cancer among Amish and Mennonite women who participated in a no-cost women’s health clinic at 26 locations throughout some of the most rural and resource-starved sections of the state. Statistically significant differences were found in total knowledge scores between Amish and Mennonite women, with Amish women having lower total knowledge scores and stronger beliefs in myths associated with breast cancer cause and symptoms. Level of education was not significantly associated with total knowledge scores, even though almost 30% of Mennonite women had completed high school or received their GED (general education development test). More research is needed on the impact of knowledge sharing among collectivistic cultures like the Amish and the role that community plays in disseminating health care knowledge and practices [14].

While almost three-fourths of the respondents stated they did not know what cancer is or what caused it, many gave scientifically accurate responses and demonstrated an understanding of cancer etiology. When responding to what causes cancer, one Amish woman stated “Cells that are inflamed and foreign to your body and can come from unhealthy habits & chemicals and environment that you’re in. But then I see somebody and ask why did it happen to them. Unhealthy cells. Besides that an ugly word that nobody wants to hear for themselves.” One Mennonite woman stated “I tend to think it’s probably genetics that predispose to something in the environment that affects the DNA in the cell.” These findings align with the responses obtained in a survey conducted among old order Amish women in Central Illinois, where “genetics/heredity” emerged as the most common answer when asked about the causes of breast cancer. Although not statistically significant, both studies identified common themes in the responses, including injuries, poor health, and environmental factors. [15].

Our study shows that sharing breast cancer risk facts may not translate into self-perceived risk. Since numeracy has been shown to be one of the best predictors in the accuracy of health-related risk factors and outcomes, future studies should explore numeracy proficiency among Amish and Mennonite communities [16]. Contrary to recent research that found people tended to overestimate national lifetime risk of breast cancer [17], the majority of women in our study underestimated risk. However, women with a family history of breast cancer were more inclined to perceive a higher risk compared to those without such history, mirroring the results from a study on health beliefs in rural Appalachia [18].

One limitation of our study is that the participants were women actively seeking healthcare at a women’s cancer clinic and as such may be much more knowledgeable and engaged in breast cancer screening activities. Unlike other limited studies surrounding knowledge and beliefs of Amish and Mennonite communities that only focused on one particular settlement, our study included women from multiple church districts throughout Ohio with varying degrees of technology adoption and access to health information. While the services provided were standardized across all clinic sites with very few cancellations or “no shows” at the clinics, access to transportation services and technology such as telephone service may have prevented some participants from contacting team members to reschedule or ask for transportation assistance in a timely manner.

This study is the largest study conducted on breast cancer knowledge with multiple Anabaptist communities. The findings showcase the importance of understanding knowledge surrounding specific health issues in order to better develop health education interventions aimed at correcting misinformation about risk and screening prevalence. Instead of sharing lifetime risks of developing or dying from breast cancer, future education interventions should share national breast cancer screening rates, which has shown to increase self-efficacy and likelihood of following recommended screening guidelines [17]. Further, convening education programs outside of the clinics in homes and at community settings will provide additional opportunities for those not scheduled or interested in seeking out health care services at one of the clinic sites.

## Data Availability

The datasets generated and analyzed during the current study are not yet publicly available as they are currently being analyzed for additional manuscripts.
